# Viral blips and hidden infections: pitfalls in HIV-1 prophylaxis by broadly neutralizing antibodies

**DOI:** 10.1038/s41392-025-02230-x

**Published:** 2025-05-09

**Authors:** Fabian Zech, Frank Kirchhoff

**Affiliations:** Institute of Molecular Virology, Ulm University Medical Center, Ulm, Germany

**Keywords:** Infectious diseases, Molecular medicine

A study published in *Nature*^1^ by Gonelli and colleagues reveals that potent, broadly neutralizing antibodies (bNAbs) delay viremic simian immunodeficiency virus (SIV) infection in rhesus macaques but do not fully prevent subclinical infections. Despite bNAb concentrations being significantly higher than supposed protective thresholds, transient viral “blips” occurred, which suggests that bNAb prophylaxis can mask subclinical infections and has implications for the interpretation of HIV-1 prevention trials.

More than 40 years after HIV-1 was identified as causative agent of AIDS, no safe and effective vaccine is available. Reasons for this are the enormous variability of HIV-1 and its ability to integrate into the host cell genome. While protection against disease is sufficient for acute viral pathogens, sterile immunity (complete protection), is critical for HIV-1, as it usually persists for life. HIV-1 employs numerous mechanisms to prevent effective immune responses. Consequently, only a small percentage of people living with HIV (PLWH) produce broadly neutralizing antibodies (bNAbs) able to neutralize diverse HIV-1 strains, and for a long time only a few effective bNAbs were available.

This has changed over the past two decades. While it remains a major challenge to generate immunogens that induce bNAbs, technical advances enabled the identification and production of many such antibodies. These bNAbs neutralize HIV-1 by targeting conserved epitopes in the viral envelope glycoprotein (Env), including the CD4-binding site (e.g., VRC01), the V2 apex loop (e.g., PG9/PGDM1400), the glycan-V3 region (e.g., PGT121/10-1074), and the gp41 membrane-proximal external region (e.g., 10E8). Their broad activity, high efficiency, and good tolerability make bNAbs promising candidates for therapeutic and prophylactic HIV-1 interventions.^2^ Like antiretroviral treatment, monotherapy with bNabs selects viral escape mutants. However, combinations of bNAbs targeting different Env domains were reported to achieve superior protective efficacy, prevent the emergence of resistant viral variants, and/or drive mutations in Env that impair virus fitness.

Most studies on bNAbs have been performed using the simian-human immunodeficiency virus (SHIV)/rhesus macaque model. These chimeric simian immunodeficiency virus (SIV) constructs contain HIV-1 Env proteins, allowing to examine the effects of bNAbs against HIV-1 strains that pose the major threat to humans. The SHIV/macaque model has also been optimized to study HIV transmission through repeated low-dose rectal or vaginal exposure, mimicking natural infection in humans. These studies have provided insights into neutralization thresholds, protection doses, and viral escape. Encouragingly, the results suggested that combinations of certain bNAbs provide complete protection when administered in sufficient quantity before SHIV exposure.^3^ However, the exact bNAb concentrations required to prevent infection remained unclear. Furthermore, the sampling frequencies in human trials do not allow precise determination of bNAb levels at the time of infection and may miss low-level transient viral replication.

To address this, Gonelli and colleagues used the SIVmac/macaque model.^1^ SIVmac is closely related to HIV-2 and both originate from SIVsmm infecting sooty mangabeys. In their initial experiment, they infused macaques with partially and/or fully neutralizing bNAbs, waited five days to allow tissue penetration, and then challenged them weekly with a Tier 2 SIVsmm molecular clone. They found that only fully neutralizing bNAb delayed virus infection and reduced peak viremia. Alarmingly, early subclinical infections occurred at nNAb plasma concentrations thought to provide effective protection. Such infections may have been missed in other studies due to infrequent sampling or assay sensitivity, as the “viral blips” are transient and associated with low viremia.

**Fig. 1:**
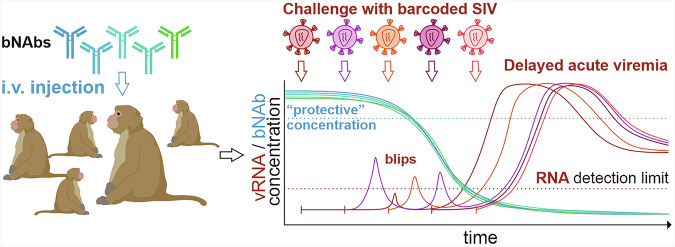
bNAbs can delay the onset of acute viremia in SIVmac-infected rhesus macaques, but subclinical infections frequently occur at high bNAb levels. Using genetically barcoded SIVmac239 constructs, Gonelli and colleagues found that viral blips - low, transient viremia - occurred while bNAb concentrations were much higher than the levels typically thought to achieve effective neutralization. Monkey images have been modified from BioRender.com

The initial experiment did not pinpoint the exact time of infection, preventing precise information on corresponding bNAb levels. To overcome this, Gonelli and colleagues used barcoded derivatives of the infectious molecular clone SIVmac239, which has been used in viral pathogenesis studies for over three decades (Fig. 1). These barcoded viruses contain neutral sequence alterations that allow precise identification of the infecting virus variant in repeated challenge experiments. Weekly rectal challenges with this difficult-to-neutralize Tier 3 virus confirmed that effective bNAbs delay infection and persistent viremia. Additionally, peak viremia was lower and developed more slowly. However, set-point viral loads were similar in treated and untreated animals, and viral blips were observed at high bNAb concentrations in most macaques.

These results raise concerns that protective effects may be overestimated because subclinical infections that may give rise to efficient viral replication and disease progression once bNAb levels decline were missed. It will be important to clarify whether subclinical infections at high bNAb concentrations also occur in the SHIV/macaque model and, most importantly, in humans. SIVmac is valuable for studies on HIV/AIDS, and its Env protein shares structural features and entry mechanisms with HIV-1. However, HIV-1 and SIVmac Envs differ significantly in glycosylation patterns, CD4-binding affinity, conformational dynamics, neutralization sensitivity, and coreceptor tropism. Furthermore, a larger number of potent bNAbs is available for HIV-1 than for SIVmac. Another key question is why bNAbs fail to protect at high plasma levels. Viral variation was not an issue since Gonelli et al. used molecular clones. Further studies are needed to assess bNAb concentrations at the site of viral entry and in lymphoid tissues representing the major site of viral replication. Gonelli and colleagues used the rectal route for challenges, which has a higher per-act risk in humans than vaginal transmission but occurs less frequently in general-population settings. It will be important to see whether similar results will be obtained in vaginal challenge models, as heterosexual transmission is the major mode of viral spread in humans. Overall, replicating the conditions of sexual HIV-1 transmission in non-human primate models is highly challenging.

Transient viral blips are also commonly observed in PLWH on cART.^4^ However, low-magnitude blips are usually not associated with virologic failure and disease progression. The results of Gonelli and colleagues indicate that high doses of effective bNAbs do not prevent infection entirely but efficiently control viral replication. As an alternative to intravenous infusion, expression of bNAbs by Adeno-Associated Virus-based vectors may allow to maintain high levels of bNAbs over long time periods.^5^ It will be of interest to clarify whether subclinical infections and transient blips, with largely controlled viral replication, are tolerated or associated with inflammation or other harmful consequences. Additionally, studies are needed to elucidate whether more complex combinations of potent bNAbs are superior to double or triple regimens, and if specific types of bNAbs are more effective in vivo, despite similar efficiency in standard neutralization assays. Notably, infrequent treatment with lenacapavir, a long-acting, potent capsid inhibitor, may provide a highly effective and safe alternative for HIV-1 pre-exposure prophylaxis.

While the development of safe and effective protective bNAb treatments against HIV-1 infection still faces challenges, significant progress has been made. Viral escape can be prevented through combinations of bNAbs targeting distinct conserved Env domains, and the low stability of bNAbs can be addressed using AAV-driven expression or Fc modifications. Key areas for further research include understanding the mechanisms of effective protection, optimizing bNAbs for action at virus entry sites, and identifying the most potent combinations. The study of Gonelli and colleagues highlights the importance of frequent sensitive monitoring of potential treatment failures and cautious interpretation of results. While further research is needed, multi-bNAb strategies, combined with mucosal-specific optimization, hold promise for real-world applicability.
